# TLR2/6 agonists and IFNγ synergize to induce melanoma cells to produce T-cell recruiting chemokines

**DOI:** 10.1186/2051-1426-1-S1-O17

**Published:** 2013-11-07

**Authors:** Ileana S Mauldin, Ena Wang, Donna H Deacon, Craig L Slingluff

**Affiliations:** 1 Surgery, University of Virginia, Charlottesville, VA, USA; 2 Transfusion Medicine, National Institutes of Health, Bethesda, MD, USA

## 

Melanoma is currently the most lethal form of skin cancer. Clinical efforts to combat melanoma have included approaches to induce the immune system, through T-cell directed therapies, to clear melanoma. These approaches require that immune cells enter the tumor microenvironment to be clinically beneficial. However, most tumors lack significant immune infiltration prior to immune therapy, and immune therapies are hindered by a persistent lack of immune cell infiltration. Chemokines can promote T-cell migration into tumors; therefore agents that induce chemokine production by melanoma cells to promote T-cell infiltration may be clinically beneficial, and could potentially work synergistically with current immune treatments for melanoma. We hypothesized that TLR ligation of melanoma would induce immune cell attracting chemokine secretion directly from melanoma cells, alone or in combination with IFNγ. To test this hypothesis, we treated human melanoma cell lines (VMM1, DM13, DM93 and DM122) individually with TLR agonists and analyzed effects on the expression of chemokines CCL2-5, CXCL9-10, and CXCL12 by flow cytometric analysis. The following TLR agonists were tested: MALP-2 and FSL-1 (TLR2/6), poly-ICLC (TLR3), LPS (TLR4), CpG (TLR9), Resiquimod (TLR7/8), Imiquimod (TLR7). Our studies found that melanoma express TLRs 2, 3, 4, 6, 7, and 9, but that without exogenous stimulation, melanoma cells produce few immune cell attracting chemokines. Furthermore, stimulation of melanoma with the TLR agonists alone induced little chemokine production. However, TLR2/6 treatment of melanoma cells (MALP-2 or FSL-1) given in combination with IFNγ resulted in significant increases in CXCL10 and CCL3 chemokine production by melanoma. We found that TLR2 and TLR6 are widely expressed on human melanoma cells. Furthermore, stimulation of fresh patient melanoma specimens with TLR2/6 agonists plus IFNγ induces CXCL10 production from melanoma cells, endothelial cells and, to a lesser extent, several immune cell subsets including dendritic, monocytic, macrophage, and B-cell. Finally, ex vivo migration assays demonstrated that stimulation of melanoma cells with TLR2/6 agonists plus IFNγ increases CD4+ and CD8+ T-cell migration toward melanoma cells. CXCL10 has been implicated as a critical chemokine supporting T-cell infiltration into the tumor microenvironment. Collectively, these data identify TLR2/6 agonists and IFNγ as key inducers of CXCL10 production by melanoma cells and suggest that for tumors lacking immune cell infiltrates, systemic immune therapies may be improved by combination with intralesional therapy using TLR2/6 agonists plus IFNγ.

